# Comparison of the Disposable Streamlined Liner of the Pharynx Airway and the Disposable I-gel in Anaesthetized, Paralyzed Adults: A Randomized Prospective Study

**DOI:** 10.1155/2015/971059

**Published:** 2015-12-01

**Authors:** Khaled EL-Radaideh, Ala"a Alhowary, Diab Bani Hani

**Affiliations:** Department of Anesthesiology, Faculty of Medicine, Jordan University of Science and Technology, P.O. Box 953, Irbid 21110, Jordan

## Abstract

*Introduction*. This study compared streamlined liner of the pharynx airway (SLIPA) and I-gel noninflatable, single-use, supraglottic airway device (SAD) performance in anesthetized, paralyzed adults.* Methods*. Eighty adults (ASA physical statuses I–III) who were undergoing elective procedures under general anesthesia with an SAD were enrolled in this prospective, randomized, single-blind study. Subjects were randomly and evenly assigned to the SLIPA or I-gel group for intraoperative airway management. Ease and number of insertions, insertion time, oropharyngeal sealing pressure, hemodynamic response, oxygen saturation (SpO_2_), end-tidal CO_2_ (EtCO_2_), and peri- and postoperative complications were examined.* Results*. The SLIPA and I-gel devices were successfully inserted in 100% and 95% of subjects, respectively. In two I-gel subjects (5%), ventilation was not possible after two attempts, but a size 55 SLIPA was successfully inserted in both cases. Forty-two and 38 patients were ultimately included in the SLIPA and I-gel groups, respectively. Insertion time was significantly shorter with the SLIPA (11.19 ± 3.03 s) than with the I-gel (15.05 ± 6.37 s, *P* = 0.003). Oropharyngeal sealing pressure was significantly higher in SLIPA (28.76 ± 3.11 cmH_2_O) than in I-gel (25.9 ± 3.65 cmH_2_O) subjects (*P* = 0.001). Blood staining occurred more frequently in SLIPA (*n* = 8, 19.0%) than in I-gel (*n* = 5, 13.2%) patients (*P* < 0.01). Heart rate, mean arterial blood pressure, SpO_2_, and EtCO_2_ were not significantly different between groups.* Conclusion*. Although blood staining incidence was higher, SLIPA insertion was easier and faster than I-gel insertion. The SLIPA provided better airway sealing pressure. Both devices had similar mechanical ventilation and oxygenation characteristics and comparable hemodynamic stability. Both noninflatable SADs are useful, but SLIPA rapid insertion and good airway sealing make it an effective alternative to the I-gel.

## 1. Introduction

Many supraglottic airway devices (SADs) are currently available for use. In the field of anesthesia, they are used during spontaneous or intermittent positive pressure ventilation. In intensive care medicine, they are a valuable rescue airway tool in emergency airway management [1–3]. These devices have become indispensable to anesthesiologists [4].

Dr. Charlie Brain first invented the inflatable cuffed laryngeal mask in the early 1980s and, since then, many relatively new SADs have been described [4, 5]. Both the I-gel (Intersurgical, Wokingham, UK) and the streamlined liner of pharyngeal airway (SLIPA, Medical Ltd., London, UK) are cuffless, anatomically preshaped, perilaryngeal sealers [6, 7]. The I-gel has a noninflatable cuff made from a gel-like thermoplastic elastomer (styrene ethylene butadiene styrene) [1]. This cuff exerts slight pressure on the pharyngolaryngeal structure, providing a perilaryngeal seal with only minimal risk of tissue compression [8]. The I-gel has a semirigid stem that acts as stabilizer within the oral cavity. This stabilizer reduces the likelihood of bad positioning, while allowing for rapid, easy, safe, and reliable application [9]. The incorporated gastric channel also facilitates gastric tube insertion, which allows for venting of gastric contents [10]. The SLIPA is a noncuffed, single-use SAD that is made of soft plastic (ethylenevinylacetate copolymer) and has a hollow chamber to capture regurgitated liquids, which potentially reduces the risk of aspiration [11].

The efficacy of noninflatable SADs, like the I-gel and SLIPA, has been previously compared with inflatable SADs, like the classic, Proseal, and supreme laryngeal mask and laryngeal tubes [12–15]. However, to the best of our knowledge, only two studies have compared the I-gel and SLIPA to each other [14, 16]. Therefore, we compared these two noninflatable SADs in a randomized, prospective clinical study, evaluating device performance in detail. Specifically, we evaluated oropharyngeal sealing pressure (OSP), the ease and speed of insertion, the success rate of insertion, the number of insertions, the hemodynamic responses induced by airway insertion (blood pressure and heart rate), oxygen saturation (SpO_2_), end-tidal carbon dioxide (EtCO_2_), and incidence of postoperative complications (e.g., traces of blood on the device, sore throat, hoarseness, and dysphagia).

## 2. Methods

This study protocol was reviewed and approved by the Institutional Review Board. All subjects provided written informed consent to participate in the study. This study was conducted according the tenets of the Declaration of Helsinki.

A total of 80 adults with an American Society of Anesthesiologists (ASA) physical statuses I–III who were scheduled for various elective procedures conducted under general anesthesia with an SAD were enrolled in this prospective, randomized single-blind study. Subjects were excluded from participation if they were younger than 18 years of age, had contraindications for laryngeal airway device use, were obese (body mass index ≥ 35), were pregnant, had a full stomach, currently had a sore throat, or were undergoing emergency surgery. Patients with neck, respiratory, or digestive tract pathology were also excluded.

No study subject was premedicated. Upon arrival to the operating room, intravenous access was established and the subject was monitored with electrocardiography, noninvasive blood pressure (NiBP) measurement, and pulse oximetry. Before inducing general anesthesia, subjects were randomly assigned in a 1 : 1 ratio to either the I-gel or the SLIPA group. Subject randomization was done using computer-generated random numbers, which were held in a series of sealed envelopes until the subject arrived in the operating room. At this point, an envelope was opened to reveal whether the subject was allocated to the I-gel (*n* = 40 subjects) or the SLIPA (*n* = 40 subjects) group.

The patient was placed in the supine position with the head placed on a jelly donut head ring. Baseline NiBP, heart rate (HR), and peripheral oxygen saturation (SpO_2_) were recorded. Following preoxygenation with 100% oxygen for 3 minutes, anesthesia was slowly induced with fentanyl (1.0–2.0 *μ*g/kg) and propofol (2.0–2.5 mg/kg) and a neuromuscular blockade was achieved with atracurium (0.5 mg/kg). Face mask ventilation was done with 100% oxygen and 2% isoflurane for ninety seconds after neuromuscular blocker injection. The assigned device was then well lubricated with a thin layer of water-based lubricant and inserted. The appropriate SAD size was selected by the anesthesiologist based on subject bodyweight and height and manufacturer guidelines. For the I-gel, size 3 was used when subject weight was less than 50 kg, size 4 was used when subject weight was between 50 and 70 kg, and size 5 was used when subject weight was more than 70 kg. For the SLIPA, sizes 47, 49, 51, and 53 were used for small, medium, medium-to-large, and large females, respectively. Sizes 53, 55, and 57 were used for small, medium, and large males, respectively.

Anesthesia was maintained with isoflurane (1.0–1.5%) in 35% oxygen and air. All subjects underwent intermittent positive pressure ventilation with a tidal volume of 7 mL/kg and a respiratory rate of 12–14 breaths per minute until the end of the procedure when they were allowed to breathe spontaneously. If necessary, residual neuromuscular blockade was reversed with neostigmine (2.5 mg) and atropine (1.0 mg). The SAD was routinely removed after the subject had regained consciousness and adequately responded to verbal commands. The removed SAD was immediately examined for the presence or absence of blood.

In addition to subject demographic data and surgical procedure duration, the following data were collected:

- Baseline mean arterial blood pressure (MAP), HR, and SpO_2_.
- MAP, HR, and SpO_2_ before anesthesia induction, before SAD insertion, and 1, 5, and 10 minutes after SAD insertion. Measurements were also made immediately after SAD removal.
- EtCO_2_ 15 minutes after the SAD insertion, using seidestream CO_2_ module of GE medical systems.
- The time taken for successful SAD insertion, which was defined as the time from SAD pick-up by the anesthesiologist to visible movement of the chest, appearance of a square wave on the capnograph trace, SpO_2_ ≥ 95% with SAD positive pressure ventilation, and the absence of leaks. All times were measured on stopwatches mounted on operating theater walls.
- The number of insertion attempts. Two insertion attempts were allowed before insertion failure was deemed to have occurred. If, after the first attempt, ventilation was inadequate, SAD position adjustment was allowed by gentle pushing or pulling of the device, chin lift, jaw thrust, head extension, or neck flexion. Adequacy of ventilation was reassessed and, if still not sufficient, the device was removed and a second insertion was attempted. If the second attempt was also unsuccessful, despite adjustment maneuvers, a SAD failure was recorded and no further data were collected from that subject for the failed device.
- Ease of airway insertion which was qualitatively evaluated using the following 4-point scale [17]: 1: easy insertion on first attempt with no need for adjustment, 2: slightly difficult insertion on first attempt with at least one adjustment maneuver needed, 3: obviously difficult insertion on the second attempt, and 4: impossible (more than 2 attempts or no SAD insertion).
- The OSP being measured once an effective airway had been achieved using a fresh gas flow rate of 5 L/min, closing the adjustable pressure limiting valve of the anesthetic circuit and recording the pressure when gas was heard leaking around the device (assessed by listening over the mouth) [18].
- Occurrence of events during SAD insertion and anesthesia (e.g., lips or dental injury, coughing, hiccup, regurgitation, and SpO_2_ < 92%).
- Asking subjects if they had a sore throat, dysphagia, voice hoarseness, tongue or jaw numbness, or neck pain thirty minutes (in recovery room) and 6 hours (in the ward) after surgery. An independent, blinded investigator interviewed subjects and recorded the presence or absence of these postoperative events.

### 2.1. Statistical Analyses

Sample size calculations were based on two previous studies comparing the SLIPA to the laryngeal mask airway (OSP = 24 ± 6 cmH_2_O) [19] and the I-gel to the laryngeal mask airway Supreme (OSP = 24.4 ± 4.3 cmH_2_O) [20]. For the primary end-point to be an interdevice OSP difference of 4 cmH_2_O, a sample size of 35 subjects per group were needed with a standard deviation of 6 cmH_2_O, an alpha error of 0.05, and a power of 80%. A total of 40 subjects were enrolled in each group to allow for potential subject dropout.

Independent (unpaired) *t*-tests were used to compare OSP, insertion time, demographic data (age, weight, and height), surgical procedure duration, and hemodynamic data (MAP and HR). The SpO_2_ and EtCO_2_ were also compared using Independent (unpaired) *t*-tests. Chi-square tests were used to compare differences between groups in success rate, insertion attempts, insertion ease, device blood presence/absence, intra- and postoperative airway morbidity, gender distribution, and ASA physical status. The Statistical Package for Social Sciences (SPSS) software (version 17 for Windows, SPSS, Inc., Chicago, IL, USA) was used to perform all statistical analyses. Unless otherwise stated, data are expressed as mean ± standard deviation. Statistical significance was defined as *P* < 0.05.

## 3. Results

Our study ultimately included 80 patients who were evenly divided between the SLIPA and I-gel groups. There were no significant differences between groups in demographic or surgical data (Table 1). In the 40 I-gel subjects, the I-gel was successfully inserted in 36 patients (90%) on the first attempt and in 2 patients (5%) on the second attempt (overall success rate = 95%). In 2 subjects (5%) adequate ventilation was not achieved after two I-gel insertion attempts. In these cases, a size 55 SLIPA was successfully inserted on the second attempt (Table 2). Data from both cases were excluded from I-gel group postinsertion analyses, but were included in SLIPA group postinsertion analyses. The SLIPA was successfully inserted in all 40 original SLIPA subjects. Including the 2 subjects from the I-gel group, the SLIPA was successfully inserted in 40 subjects (95.2%) on the first attempt and in 2 subjects (4.8%) on the second attempt (overall success rate = 100%, Table 2).

**Figure 1:**
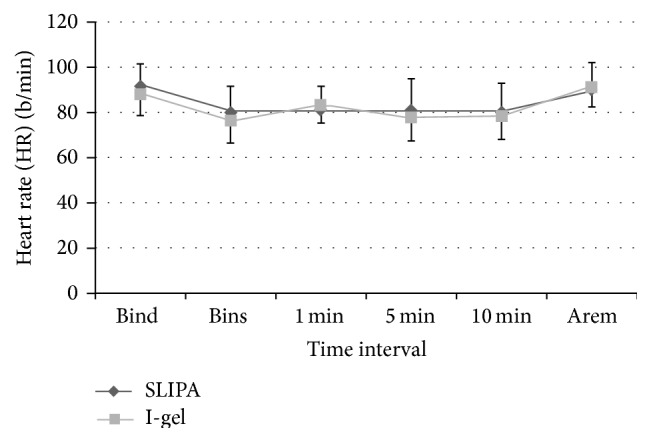
Heart rate before and after supraglottic airway device inserion. Data collected after device removal are also shown. Mean values are presented and error bars represent one standard deviation. SLIPA: streamlined liner of pharyngeal airway, Bind: before induction, and Bins: before insertion. Time 0 was defined as the time at which the device was inserted. 1, 5, and 10 minutes after insertion. Arem: immediately after removal of the airway device.

**Figure 2:**
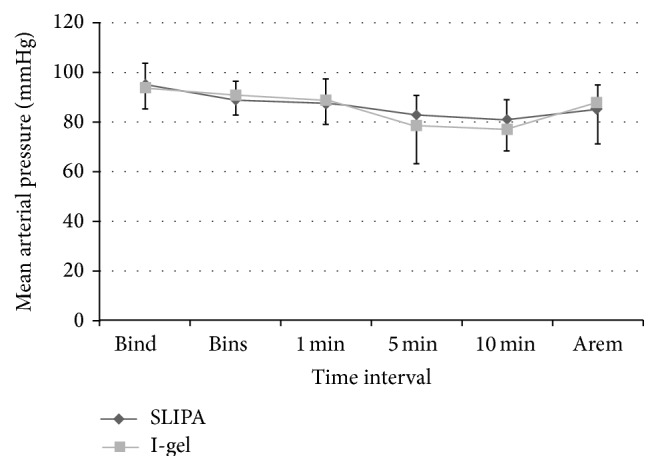
Mean arterial pressure before and after supraglottic airway device insertion. Data collected after device removal are also shown. Mean values are presented and error bars represent one standard deviation. SLIPA: streamlined liner of pharyngeal airway, Bind: before induction, and Bins: before insertion. Time 0 was defined as the time at which the device was inserted. 1, 5, and 10 minutes after insertion. Arem: immediately after removal of the airway device.

Anesthesiologists rated SLIPA insertion as easier than I-gel insertion. An effective airway was achieved on the first attempt without performing adjustment maneuvers in 88.1% of SLIPA subjects and 80.0% of I-gel subjects (*P* < 0.02, Table 2). Insertion times for successful cases were significantly shorter in the SLIPA group. Additionally, OSP was significantly higher following SLIPA insertion (Table 3). There were no significant differences between the groups in SpO_2_ or EtCO_2_ (Table 4). Both groups also had similar HR and MAP at all time points examined (Figures 1 and 2). No adverse events occurred during surgery in any subject.

Upon device removal, blood staining was more frequently detected in the SLIPA group including the 2 cases enrolled from the I-gel group than in the I-gel group. However, intra- and postoperative airway morbidity rates were not significantly different between groups (Table 5). In the SLIPA group, 3 subjects complained of a sore throat and one patient complained of dysphagia. In the I-gel group, 6 subjects complained of a sore throat and 2 subjects complained of dysphagia (Table 5).

## 4. Discussion

The I-gel is an innovative second-generation SAD that was introduced by Intersurgical in 2007 in three adult sizes. In 2010, the company introduced four pediatric sizes. After gently pressing down the chin, the leading I-gel soft tip should be introduced into the patient's mouth in a direction towards the hard palate. The device should then be glided downward and backward along the hard palate with continuous force until a definitive resistance is felt [21–23]. The SLIPA is inserted by sliding the SAD over the tongue, lifting the tongue with it, as is done with a laryngoscope [24].

Both the SLIPA and I-gel devices were easily inserted with high success rates in our study (95.2% and 90.0% on the first attempt, resp., and 100% and 95% overall success rate, resp.). The insertion of the I-gel was unsuccessful in 2 subjects because of insufficient airway sealing despite adjusted airway size. Both of these subjects subsequently underwent successful SLIPA insertion. Trivedi and Patil [25] reported having a similar experience. They compared the I-gel to the Proseal laryngeal mask (PLMA) in 60 patients (30 patients each group). The I-gel was successfully inserted on the first try in 26 patients (86.7%) and on the second attempt in 2 patients (overall success = 93.3%). The remaining 2 patients (6.7%) required endotracheal tube insertion because of 2 failed attempts to secure the airway with the I-gel.

Jeon et al. [26] conducted a prospective, randomized study comparing the PLMA and I-gel devices in 30 women undergoing gynecological laparoscopy. They reported a success rate of 100% with both devices on the first insertion attempt. Their findings were consistent with other studies, which reported insertion success rates of 84–100% for the I-gel [5, 14, 27–29] and 96–100% for the SLIPA [30–32]. Despite its high insertion success rate, we found that the I-gel was more difficult to insert than the SLIPA. This result is consistent with another study [33]. The I-gel insertion difficulties stem from the insertion path that must be taken. The SAD must pass the teeth and tongue, forcing the anesthesiologist to take an insertion approach that is slightly off of the midline. Depressing the tongue with the thumb can ease the insertion process and has been advocated [33, 34].

We found that OSP with the SLIPA was 28.76 ± 3.11 cmH_2_O, which is in agreement with previous reports [31, 32]. Miller and Camporota [13] found a slightly higher OSP in a study comparing the SLIPA, the PLMA, and standard endotracheal intubation performed for laparoscopic gynecological surgical procedures. In that study no significant difference was observed in OSP obtained with the SLIPA and the PLMA. Lower OSP values than that obtained here have also been observed in previous studies comparing the SLIPA to a laryngeal mask (LMA) or the PLMA [19, 35]. The differences between studies may have resulted from the use of a muscle relaxant prior to airway device insertion [30, 32]. Muscle relaxants suppress unwanted reflexes, making it easier to insert an airway device and shortening insertion time.

Our study data showed that OSP obtained with the SLIPA was higher (28.76 ± 3.11 cmH_2_O) than that obtained with the I-gel (25.92 ± 3.65 cmH_2_O, *P* = 0.001). This difference was statistically significant and suggests that the SLIPA provides a more effective airway for positive pressure ventilation. This result is consistent with earlier studies, which found that the I-gel provides an OSP of 19–33 cmH_2_O [9, 36, 37]. Additionally, Amini and Khoshfetrat [38] and Singh et al. [39] showed that the Solus LMA and the PLMA, respectively, had a higher sealing pressure and a better esophageal seal than the I-gel. Schmidbauer et al. [40] also found that the OSP of the PLMA was higher than that of the I-gel. However, Shin et al. [41] observed no difference in OSP between the I-gel and the PLMA in adult patients. This discrepancy may have resulted from the presence of a dorsal cuff, which is found on size 3 and above PLMAs. In contrast, Goyal et al. [42] found that OSP with the I-gel was significantly higher than that with the PLMA. Because their study used small size PLMAs (size 2.5), the higher sealing pressure of the I-gel may have resulted from the absence of a dorsal cuff [43].

Our investigation revealed that, on average, SLIPA insertion time was shorter than I-gel insertion time. This finding is in agreement with a previous study [44], which showed a shorter insertion time with the SLIPA than with the Softseal LMA. Puri et al. [35] found that inserting the LMA took slightly longer than inserting the SLIPA. Oh et al. [5] compared the clinical efficacy of the SLIPA and an LMA when inserted by novice personnel with no experience with either device. They also found a shorter mean insertion time with the SLIPA than with the LMA. In contrast, Choi et al. [30] found that SLIPA insertion took longer and was more difficult than PLMA insertion. The authors attributed their finding to inexperience with the SLIPA device and difficulty in selecting the appropriate device size.

In this study the anesthesiologist inserting supraglottic airway devices had different experiences with the use of the SLIPA as well as with the I-gel.

While the principal investigator has much experience at SLIPA and I-gel insertion, some anesthesiologists investigators have comparatively little experience with using I-gel and SLIPA.

These differences between the anesthesiology staff in this respect might have altered the outcome of the study.

Our I-gel insertion time of 15 s is in agreement with the 15.62 s found by Atef et al. [29]. In their study, I-gel insertion time was shorter than that for a classic LMA. Suhitharana et al. [20] also showed comparable data for I-gel placement time. However, placement time reported by several previous studies was much shorter [14, 45, 46]. In other studies comparing the I-gel and different types of LMAs, I-gel insertion time was much longer than that observed here in both children and adults. This was true even when the time required for I-gel insertion was higher or lower than that needed for LMA insertion [47–50]. This may have resulted from additional time needed to inflate the PLMA cuff after its insertion [51], but the bulky I-gel shape may have also contributed to longer insertion times compared to the Supreme LMA [52].

With the exception of blood staining, which occurred more frequently with the SLIPA (19%) than with the I-gel (13%), we found a similar incidence of postoperative airway morbidities for both devices. These results are in agreement with Lange et al. [19], who found traces of blood on the SLIPA in 20% of patients. Puri et al. [35] compared the SLIPA and the classic LMA in 100 patients and showed that blood staining was more frequently associated with the SLIPA (40%) than with the classic LMA (12%). Choi et al. [30] also found a higher incidence of blood staining than that reported here. However, Li et al. [53] reported a lower incidence of blood staining on the SLIPA (5%) after using two different device sizing methods in 100 cases. The rigidity of the SLIPA, particularly its toe stiffness, may have resulted in the traces of blood. Many studies have also compared I-gel and PLMA blood staining incidences in children and adults. The presence of blood on the I-gel, reported as 0–10%, was much lower than that reported for the PLMA and other masks (e.g., supreme and classic LMA) [49, 54, 55]. These differences may have resulted from device material differences. The softer, more malleable I-gel material [20] may not have caused injury to the lingual and recurrent laryngeal nerves that may be caused by the fully inflated PLMA cuff [31, 32, 56]. In support of this idea, Soliveres et al. [57] found that PLMA use resulted in higher incidences of sore throat (28.6%) and dysphagia (25%) than I-gel use (3.4% and 3.4%, resp.).

The stability of hemodynamic parameters following SAD insertion and throughout surgical procedures was comparable between the SLIPA and I-gel study groups. We found no significant differences between groups in SpO_2_ or EtCO_2_ throughout the duration of surgery. Atef et al. [29] also reported hemodynamic stability with both LMA and I-gel devices, with no statistically significant differences between groups. Jindal et al. [14] examined hemodynamic changes during LMA, SLIPA, or I-gel insertion in 75 adult patients. They found no significant changes in HR or differences between the SLIPA and I-gel groups. Hemodynamic stability may have resulted from the lack of laryngoscope use during SAD insertion. Furthermore, the absence of an inflatable cuff on the SLIPA and I-gel devices could have played a large role in attenuated hemodynamic responses [58, 59].

In conclusion, insertion of the SLIPA is easier and faster than insertion of the I-gel. The SLIPA also provides a higher airway sealing pressure. However, this device is associated with a higher incidence of blood staining than other SADs. The two devices examined here have similar mechanical ventilation and oxygenation characteristics and result in comparable patient hemodynamic stability. Therefore, both noninflatable SADs are good options when choosing an airway device. Because of its rapid insertion and high quality airway seal, the SLIPA is an effective alternative SAD.

## Figures and Tables

**Table 1:** Subject demographic and surgical data.

Group	SLIPA (*n* = 40)	I-gel (*n* = 40)	*P* value
Age, years	47.15 ± 19	39.5 ± 13.59	0.460
Gender (M : F)	18/22	13/27	0.251
Height, cm	167.5 ± 8.0	165.6 ± 9.2	0.324
Weight, kg	76.95 ± 13.41	75.50 ± 14.75	0.509
ASA status (I/II/III)	19/13/8	29/9/2	0.168
Surgery duration, min	35.0 ± 15.76	27.4 ± 9.98	0.021
Type of surgery			
General surgery	9	7	
Gynecology	21	22	
Urology	7	11	
Ophthalmology	3	0	

Data are presented as mean ± standard deviation where applicable. ASA: American Society of Anesthesiologists, M: male, F: female, and SLIPA: streamlined liner of pharyngeal airway.

**Table 2:** Success rate and ease of supraglottic airway device insertion.

Group	SLIPA (*n* = 42)	I-gel (*n* = 40)	*P* value
Number of insertion attempts (*n*)			
First attempt, *n* (%)	40 (95.2%)	36 (90.0%)	*P* < 0.02
Second attempt, *n* (%)	2 (4.8%)	2 (5.0%)	
Failed to insert, *n* (%)	0 (0%)	2 (5.0%)	
Overall success rate, *n* (%)	42 (100%)	38 (95.0%)	
Ease of insertion			
Easy, *n* (%)	37 (88.1%)	32 (80.0%)	*P* < 0.001
Slightly difficult, *n* (%)	3 (7.1%)	4 (10.0%)	
Obviously difficult, *n* (%)	2 (4.8%)	2 (5.0%)	
Impossible, *n* (%)	0 (0%)	2 (5.0%)	

Data are expressed as number of patients. Easy: successful insertion on first attempt with no need for adjustment, slightly difficult: successful insertion on first attempt with the need for at least one adjustment, obviously difficult: successful insertion on second attempt, and impossible: insertion not successful after two attempts. SLIPA: streamlined liner of pharyngeal airway.

**Table 3:** Oropharyngeal sealing pressure, insertion time, and device size.

Group	SLIPA (*n* = 42)	I-gel (*n* = 38)	*P*-value
Oropharyngeal sealing pressure, cmH_2_O	28.76 ± 3.11	25.92 ± 3.65	0.001
Time needed for insertion, s	11.19 ± 3.03	15.05 ± 6.37	0.003
Size of SAD used			
SLIPA (49/51/53/55*∖*57), *n*	2/9/12/12/7	—	—
I-gel (3/4/5), *n*	—	7/23/8	—

Data are presented as mean ± standard deviation where applicable. SLIPA: streamlined liner of pharyngeal airway, SAD: supraglottic airway device.

**Table 4:** Oxygen saturation (SpO_2_) and end-tidal carbon dioxide (EtCO_2_) before and after supraglottic airway device insertion.

	SLIPA (*n* = 42)	I-gel (*n* = 38)	*P* value
SpO_2_ (%)			
Before anesthesia induction	97.3 ± 1.73	98.0 ± 1.52	*P* > 0.05
Before device insertion	98.52 ± 1.48	97.05 ± 2.31	*P* > 0.05
1 min after device insertion	98.14 ± 1.53	97.92 ± 1.36	*P* > 0.05
5 min after device insertion	99.14 ± 0.98	99.05 ± 0.84	*P* > 0.05
10 min after device insertion	99.12 ± 0.89	99.03 ± 0.82	*P* > 0.05
2 min after device removal	96.64 ± 1.55	96.08 ± 2.05	*P* > 0.05
EtCO_2_ (mmHg)			
15 min after device insertion	36.6 ± 4.1	35.8 ± 2.7	*P* > 0.05

Data are presented as mean ± standard deviation. SLIPA: streamlined liner of the pharynx airway.

**Table 5:** Incidence of postoperative complications related to intubation and airway morbidity.

	SLIPA (*n* = 42)	I-gel (*n* = 38)	*P* value
Blood on SAD, *n* (%)	8 (19.0%)	5 (13.2%)	*P* < 0.01
Sore throat, *n* (%)			
1 hr after device removal	3 (7.1%)	6 (15.8%)	*P* > 0.05
8 hr after device removal	0 (0%)	2 (5.3%)	*P* > 0.05
Dysphagia, *n* (%)			
1 hr after device removal	1 (2.4%)	2 (5.3%)	*P* > 0.05
8 hr after device removal	0 (0%)	2 (5.3%)	*P* > 0.05
Hoarseness, *n* (%)	0 (0%)	0 (0%)	*P* > 0.05

SLIPA: streamlined liner of pharyngeal airway, SAD: supraglottic airway device.

## References

[1] Lee K. H., Kang E. S., Jung J. W., Park J. H., Choi Y. G. (2013). Use of the i-gel supraglottic airway device in a patient with subglottic stenosis—a case report. *Korean Journal of Anesthesiology*.

[2] Ueki R., Komasawa N., Nishimoto K., Sugi T., Hirose M., Kaminoh Y. (2014). Utility of the Aintree Intubation Catheter in fiberoptic tracheal intubation through the three types of intubating supraglottic airways: a manikin simulation study. *Journal of Anesthesia*.

[3] Gruber C., Nabecker S., Wohlfarth P. (2013). Evaluation of airway management associated hands-off time during cardiopulmonary resuscitation: a randomised manikin follow-up study. *Scandinavian Journal of Trauma, Resuscitation and Emergency Medicine*.

[4] Sardi A. S., Britto M., Rangel J. (2013). Comparison of postoperative throat and neck complaints after the use of the i-gel versus the traditional laryngeal mask. *Open Journal of Anesthesiology*.

[5] Oh S.-K., Lim B. G., Kim H., Lim S. H. (2012). Comparison of the clinical effectiveness between the streamlined liner of pharyngeal airway (SLIPA) and the laryngeal mask airway by novice personnel. *Korean Journal of Anesthesiology*.

[6] Gibbison B., Cook T. M., Seller C. (2008). Case series: protection from aspiration and failure of protection from aspiration with the i-gel airway. *British Journal of Anaesthesia*.

[7] Miller D. M. (2004). A proposed classification and scoring system for supraglottic sealing airways: a brief review. *Anesthesia and Analgesia*.

[8] Singh J., Yadav M. K., Marahatta S. B., Shrestha B. L. (2012). Randomized crossover comparison of the laryngeal mask airway classic with i-gel laryngeal mask airway in the management of difficult airway in post burn neck contracture patients. *Indian Journal of Anaesthesia*.

[9] Gatward J. J., Cook T. M., Seller C. (2008). Evaluation of the size 4 i-gel airway in one hundred non-paralysed patients. *Anaesthesia*.

[10] Foucher-Lezla A., Lehousse T., Monrigal J. P., Granry J. C., Beydon L. (2013). Fibreoptic assessment of laryngeal positioning of the paediatric supraglottic airway device i-gel. *European Journal of Anaesthesiology*.

[11] Luba K., Cutter T. W. (2010). Supraglottic airway devices in the ambulatory setting. *Anesthesiology Clinics*.

[12] Abd Rahman I., Nurlia Y., Wan Rahiza W., Esa K., Nadia M., Raha A. (2012). Comparison between the use of LMA and SLIPA in patients undergoing minor surgeries. *Journal of Surgical Academia*.

[13] Miller D. M., Camporota L. (2006). Advantage of ProSeal and SLIPA airways over tracheal tubes for gynecological laparoscopies. *Canadian Journal of Anesthesia*.

[14] Jindal P., Rizvi A., Sharma J. P. (2009). Is I-gel a new revolution among supraglottic airway devices?—a comparative evaluation,. *Middle East Journal of Anesthesiology*.

[15] Russo S. G., Cremer S., Eich C. (2012). Magnetic resonance imaging study of the *in vivo* position of the extraglottic airway devices i-gel and LMA-Supreme in anaesthetized human volunteers. *British Journal of Anaesthesia*.

[16] Jackson K. M., Cook T. M. (2007). Evaluation of four airway training manikins as patient simulators for the insertion of eight types of supraglottic airway devices. *Anaesthesia*.

[17] El-Radaideh K., Amarin Z., Rashdan Y., Rabadi D., Khraise W., Omari M. (2011). The perilaryngeal airway and the laryngeal tube in short ophthalmic procedures in adults: a prospective randomized comparative study. *Journal of Anesthesia & Clinical Research*.

[18] Roberts M., Mani M., Wilkes A., Flavell E., Goodwin N. (2012). A randomised crossover study comparing the disposable laryngeal mask airway Supreme with the laryngeal mask airway Proseal in unparalysed anaesthetised patients. *The Internet Journal of Anesthesiology*.

[19] Lange M., Smul T., Zimmermann P., Kohlenberger R., Roewer N., Kehl F. (2007). The effectiveness and patient comfort of the novel streamlined pharynx airway liner (SLIPA) compared with the conventional laryngeal mask airway in ophthalmic surgery. *Anesthesia & Analgesia*.

[20] Suhitharan T., Teoh W. H. L. (2013). Use of extraglottic airways in patients undergoing ambulatory laparoscopic surgery without the need for tracheal intubation. *Saudi Journal of Anaesthesia*.

[21] Helmy A. M., Atef H. M., El-Taher E. M., Henidak A. M. (2010). Comparative study between I-gel, a new supraglottic airway device, and classical laryngeal mask airway in anesthetized spontaneously ventilated patients. *Saudi Journal of Anaesthesia*.

[22] I-gel User-guide http://www.i-gel.com/igel-for-anaesthesia.

[23] Siddiqui A. S., Ahmed J., Siddiqui S. Z., Haider S., Raza S. A. (2012). New single use supraglottic airway device with non-inflatable cuff and gastric tube channel. *Journal of the College of Physicians and Surgeons Pakistan*.

[24] Kim S. H., Choi E. M., Chang C. H., Kim H. K., Chung M. H., Choi Y. R. (2011). Comparison of the effect-site concentrations of remifentanil for streamlined liner of the pharynx airway (SLIPA) versus laryngeal mask airway SoftSeal insertion during target-controlled infusion of propofol. *Anaesthesia and Intensive Care*.

[25] Trivedi V., Patil B. (2009). A clinical comparative study of evaluation of Proseal LMA V/S I-GEL for ease of insertion and hemodynamic stability; a study of 60 cases. *The Internet Journal of Anesthesiology*.

[26] Jeon W. J., Cho S. Y., Baek S. J., Kim K. H. (2012). Comparison of the proseal LMA and intersurgical I-gel during gynecological laparoscopy. *Korean Journal of Anesthesiology*.

[27] Donaldson W., Abraham A., Deighan M., Michalek P. (2011). I-gel vs. AuraOnce laryngeal mask for general anaesthesia with controlled ventilation in paralyzed patients. *Biomedical Papers of the Medical Faculty of the University Palacky, Olomouc, Czechoslovakia*.

[28] Halwagi A. E., Massicotte N., Lallo A. (2012). Tracheal intubation through the I-gel supraglottic airway versus the LMA Fastrach: a randomized controlled trial. *Anesthesia and Analgesia*.

[29] Atef H. M., Helmy A. M., El-Taher E. M., Henidak A. M. (2012). Comparative study between I-gel, a new supraglottic airway device, and classical laryngeal mask airway in anesthetized spontaneously ventilated patients. *Middle East Journal of Anesthesiology*.

[30] Choi Y. M., Cha S. M., Kang H. (2010). The clinical effectiveness of the streamlined liner of pharyngeal airway (SLIPA) compared with the laryngeal mask airway ProSeal during general anesthesia. *Korean Journal of Anesthesiology*.

[31] Woo Y. C., Cha S. M., Kang H. (2011). Less perilaryngeal gas leakage with SLIPATM than with LMA-ProSealTM in paralyzed patients. *Canadian Journal of Anesthesia*.

[32] Abdellatif A. A., Ali M. A. (2011). Comparison of streamlined liner of the pharynx airway (SLIPATM) with the laryngeal mask airway ProSealTM for lower abdominal laparoscopic surgeries in paralyzed, anesthetized patients. *Saudi Journal of Anaesthesia*.

[33] Theiler L., Gutzmann M., Kleine-Brueggeney M., Urwyler N., Kaempfen B., Greif R. (2012). I-gel*™* supraglottic airway in clinical practice: a prospective observational multicentre study. *British Journal of Anaesthesia*.

[34] Theiler L. G., Kleine-Brueggeney M., Kaiser D. (2009). Crossover comparison of the laryngeal mask supreme and the i-gel in simulated difficult airway scenario in anesthetized patients. *Anesthesiology*.

[35] Puri G. D., Hegde H. V., Jayant A., Bhukal I. (2008). Haemodynamic and bispectral index response to insertion of the Streamlined Liner of the Pharynx Airway (SLIPA): comparison with the laryngeal mask airway. *Anaesthesia and Intensive Care*.

[36] Richez B., Saltel L., Banchereau F., Torrielli R., Cros A. M. (2008). A new single use supraglottic airway device with a noninflatable cuff and an esophageal vent: an observational study of the i-gel. *Anesthesia & Analgesia*.

[37] Bamgbade O. A., Macnab W. R., Khalaf W. M. (2008). Evaluation of the i-gel airway in 300 patients. *European Journal of Anaesthesiology*.

[38] Amini S., Khoshfetrat M. (2010). Comparison of the Intersurgical Solus laryngeal mask airway and the i-gel supralaryngeal device. *Anaesthesia*.

[39] Singh I., Gupta M., Tandon M. (2009). Comparison of clinical performance of I-gel with LMA-ProsealTM in elective surgeries. *Indian Journal of Anaesthesia*.

[40] Schmidbauer W., Bercker S., Volk T., Bogusch G., Mager G., Kerner T. (2009). Oesophageal seal of the novel supralaryngeal airway device I-Gel in comparison with the laryngeal mask airways Classic and ProSeal using a cadaver model. *British Journal of Anaesthesia*.

[41] Shin W.-J., Cheong Y.-S., Yang H.-S., Nishiyama T. (2010). The supraglottic airway I-gel in comparison with ProSeal laryngeal mask airway and classic laryngeal mask airway in anaesthetized patients. *European Journal of Anaesthesiology*.

[42] Goyal R., Shukla R. N., Kumar G. (2012). Comparison of size 2 i-gel supraglottic airway with LMA-proSeal and LMA-classic in spontaneously breathing children undergoing elective surgery. *Paediatric Anaesthesia*.

[43] Mitra S., Das B., Jamil S. N. (2012). Comparison of size 2.5 i-gel with proseal LMA in anaesthetised, paralyzed children undergoing elective surgery. *North American Journal of Medical Sciences*.

[44] Hein C., Owen H., Plummer J. (2006). Randomized comparison of the SLIPA (Streamlined Liner of the Pharynx Airway) and the SS-LM (Soft Seal Laryngeal Mask) by medical students. *Emergency Medicine Australasia*.

[45] Russo S. G., Cremer S., Galli T. (2012). Randomized comparison of the i-gel, the LMA Supreme, and the Laryngeal Tube Suction-D using clinical and fibreoptic assessments in elective patients. *BMC Anesthesiology*.

[46] Chauhan G., Nayar P., Seth A., Gupta K., Panwar M., Agrawal N. (2013). Comparison of clinical performance of the I-gel with LMA proseal. *Journal of Anaesthesiology Clinical Pharmacology*.

[47] Kapoor S., Jethava D. D., Gupta P., Jethava D., Kumar A. (2014). Comparison of supraglottic devices i-gel and LMA Fastrach as conduit for endotracheal intubation. *Indian Journal of Anaesthesia*.

[48] Sanuki T., Sugioka S., Komasawa N., Ueki R., Kaminoh Y., Kotani J. (2014). Comparison of insertion of the modified i-gel airway for oral surgery with the LMA flexible: a manikin study. *Anesthesia Progress*.

[49] Jadhav P. A., Dalvi N. P., Tendolkar B. A. (2015). I-gel versus laryngeal mask airway-proseal: comparison of two supraglottic airway devices in short surgical procedures. *Journal of Anaesthesiology Clinical Pharmacology*.

[50] Saran S., Mishra S. K., Badhe A. S., Vasudevan A., Elakkumanan L. B., Mishra G. (2014). Comparison of i-gel supraglottic airway and LMA-ProSeal in pediatric patients under controlled ventilation. *Journal of Anaesthesiology Clinical Pharmacology*.

[51] Kini G., Devanna G. M., Mukkapati K. R., Chaudhuri S., Thomas D. (2014). Comparison of I-gel with proseal LMA in adult patients undergoing elective surgical procedures under general anesthesia without paralysis: a prospective randomized study. *Journal of Anaesthesiology Clinical Pharmacology*.

[52] Kim H., Lee J. Y., Lee S. Y., Park S. Y., Lee S. C., Chung C. J. (2014). A comparison of i-gel and LMA Supreme in anesthetized and paralyzed children. *Korean Journal of Anesthesiology*.

[53] Li Y., Xie Y., Wei X. (2013). A novel method for SLIPA size selection, for adult patients, on the basis of chamber length. *Journal of Anesthesia*.

[54] Kim M.-S., Lee J. H., Han S. W., Im Y. J., Kang H. J., Lee J.-R. (2015). A randomized comparison of the i-gel with the self-pressurized air-Q intubating laryngeal airway in children. *Paediatric Anaesthesia*.

[55] Pant D., Koul A., Sharma B., Sood J., Lerman J. (2015). A comparative study of Laryngeal Mask Airway size 1 vs i-gel size 1 in infants undergoing daycare procedures. *Pediatric Anesthesia*.

[56] Brimacombe J., Clarke G., Keller C. (2005). Lingual nerve injury associated with the ProSeal laryngeal mask airway: a case report and review of the literature. *British Journal of Anaesthesia*.

[57] Soliveres J., Balaguer J., Richart M. T., Sánchez J., Solaz C. (2010). Airway morbidity after use of the laryngeal mask airway: LMA ProSeal vs. I-gel. *European Journal of Anaesthesiology*.

[58] Maharjan S. K. (2013). I-gel for positive pressure ventilation. *Journal of the Nepal Medical Association*.

[59] Ismail S. A., Bisher N. A., Kandil H. W., Mowafi H. A., Atawia H. A. (2011). Intraocular pressure and haemodynamic responses to insertion of the i-gel, laryngeal mask airway or endotracheal tube. *European Journal of Anaesthesiology*.

